# Multi-input/output alarming system for patients with inattention caused by higher cortical function disorder

**DOI:** 10.1186/1475-925X-12-104

**Published:** 2013-10-12

**Authors:** Abbas Orand, Sayaka Okamoto, Yutaka Tomita, Hiroyuki Miyasaka, Genichi Tanino, Shigeru Sonoda

**Affiliations:** 1 Department of Rehabilitation, Fujita Memorial Nanakuri Institute, Fujita Health University, 1865 Hisai-Isshiki-cho, Tsu, Mie 514-1296, Japan; 2 Department of Rehabilitation Medicine II, School of Medicine, Fujita Health University, 424-1 Oodoricho, Tsu, Mie 514-1295, Japan

**Keywords:** Hemiplegic, Neglect, Wireless, Instrumentation, Alarming system

## Abstract

**Background:**

To apply advanced methods of communication, sensing, and instrumentation technologies to make a system that can help patients suffering from hemispatial neglect caused by higher cortical function disorder.

**Method:**

By using several sensors and actuators, the objective was to construct a tailor-made system for each patient. The input part of the system consists of sensors, an interface and transmitters. The output part consists of a receiver, logical arithmetic, an output interface and actuators. The information from the input part is sent to the output part in a wireless manner allowing the mobility of the input and output parts.

**Results:**

The system and its functionality were realized. Voice alarming and neck muscle stimuli were applied to two patients. We could verify the applicability of the system to remind the patients to put on their wheelchair’s brake and raise its footrest before attempting to stand for transferring to their beds.

**Conclusion:**

The designed and constructed multi-input/output system can be used effectively to alarm the patients.

## Background

The application of engineering and robots in medicine and rehabilitation is becoming widespread and important. Hearing aid devices assist the individuals with hearing impairment. Electrically powered assisted legs help the hemiplegic [1] and the patients with spinal cord injury [2] to walk again. Similarly, engineering has been applied for the treatment and rehabilitation of the hemiplegic patients with inattention such as hemispatial neglect caused by higher cortical function disorder since 1940 [3] by the vestibular stimulation of the patients [4]. Other methods of training and rehabilitation such as visual scanning training [5,6] limb activation [1], repetitive transcranial magnetic stimulation [7], sustained attention training [8], optokinetic [9-11], neck muscle vibration [12,13], and trunk rotation [14] were applied afterwards in several studies [3].

The inattention syndrome can be seen in wide varieties. For example, attention impairment [15-17] can be seen in some individuals of all ages. Such patients are described as having “minimal brain dysfunction” [15] with some similarities to patients with evident central nervous system injuries. The inattention in these individuals is a significant component of the disorder. The inattention disorder can also be seen in patients with sensory neglect. Some treatments [18] target at improving spatial neglect using external sources of stimuli [11,12,14,19-24] such as visual, tactile and auditory ones [25].

Most of the attention training (sustained, alternating, selective and divided) processes are based on the improvement of the attention abilities [26] because neglect is partly due to the reduced maintenance of attention during the exploration of space [5]. The functional activities of these trainings combine auditory and visual activities requiring the activation of many different cognitive processes such as listening for descending number sequences on auditory attention tapes [26].

However, a single rehabilitation/training output stimulation has been used for all patients in the majority of the researches. Combination of output stimulations and input modalities that can suit the needs of each patient is not explored. To address this issue, we purpose a multi-stimulating output based on multi-inputs. In this study, our objective was to design a system to rehabilitate and to assist patients with inattention based on getting some inputs from the patient and output some stimuli, accordingly. We believe that a tailor-made multi-input and multi-output system is indispensable for the rehabilitation of each patient with inattention disorder, because of the wide variety of mechanisms and symptoms of the inattention [15,27,28]. Such system can be practical for the rehabilitation of the hemiplegic patients.

In order to evaluate the effect of the system, we focused on the transfer activity from the wheelchairs to the bed in patients with both physical disability and hemispatial neglect. These patients who use a wheelchair for their daily activities need to put on the wheelchair’s brakes and raise its footrests before standing to transfer to their beds. Bypassing some of these steps results in their falls.

In general, the functional activities and trainings that are used for the treatment/rehabilitation of the patients are multifaceted requiring various external stimuli. Therefore, a tailor-made system based on the individual’s / patient’s need is required. Such a system should work based on the inputs from the patients. As a result, a multi-input/output system is desirable.

## Methods

### System concept

**Figure 1:**
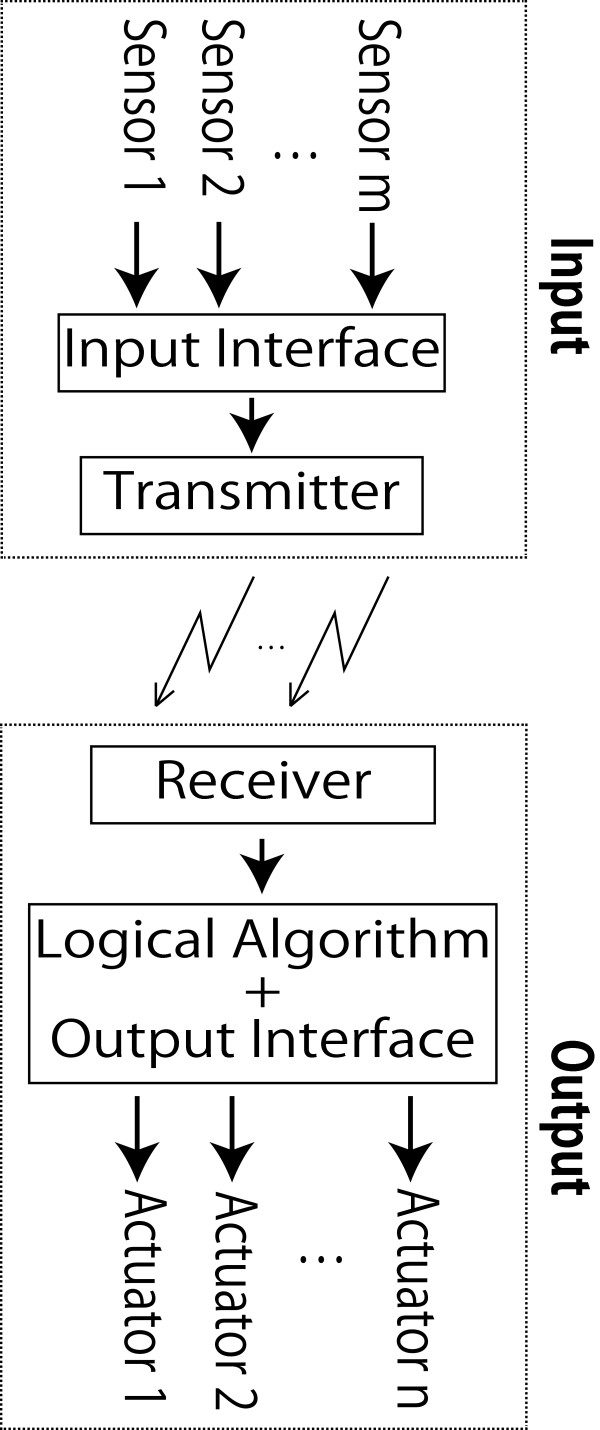
The system lay-out.

We developed a new system which consists of multi-input ‘input part’ and multi-output ‘output part’. The signal is fed into a transmitter(s) via an input interface and is sent to actuators via a receiver, a logical arithmetic and an output interface as it is shown in Figure 1. Different sensors are used to explore the situation around the patient and to discover the status of the patient. The analog signals from these sensors are converted to digital signals in the input interface for wireless transfer to the receiver. Based on the logical arithmetic and the output interface, the digital signal is converted to analog to drive an actuator(s). These actuators are then used for the output of different stimuli.

**Figure 2:**
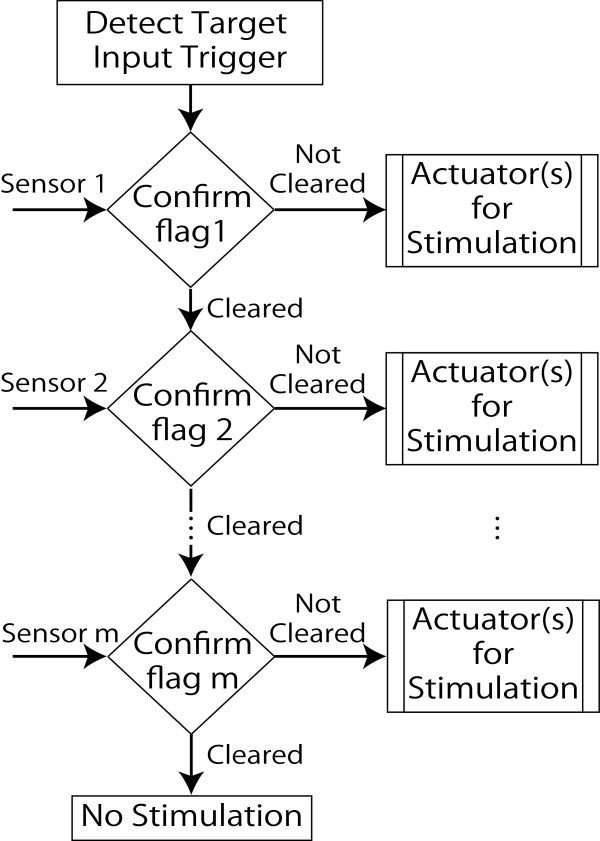
System’s logical arithmetic.

The logical arithmetic of the system is shown in Figure 2. Upon the trigger of the target input, the arithmetic starts to check for other flags statuses. In case that all of the flags are clear, actuators produce no stimulation. If any of the flags not be cleared, the actuator gives required stimulation to remind the patient.

### Transfer alarming system

**Figure 3:**
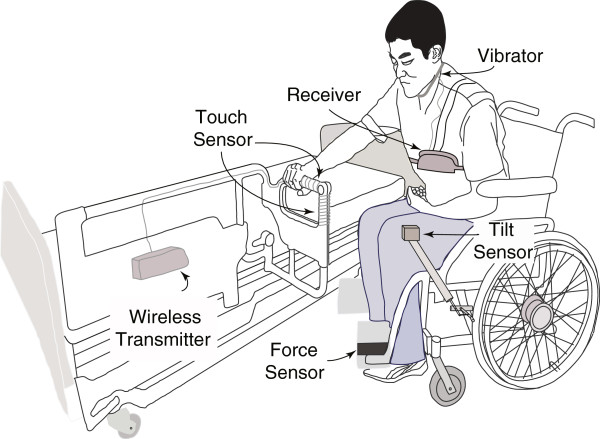
**The transfer alarming system consisting of sensors (touch, tilt, and force), a transmitters and a receiver is shown.** The small box that the patient carries includes the wireless receiver, circuitry and actuator to stimulate neck muscle. The 1^st^ transmitter mounted on the bedrail, transmits the signal from the touch sensor. The 2^nd^ transmitter (not shown) is mounted at the back of the wheelchair and is connected to the tilt and force sensors transmitting sensors’ signal to the receiver. Tilt sensor is mounted in a small box which is placed at the tip of the extending bar connected to wheelchair’s brake. Force sensor is attached to the footrest.

In order to make the transfer of the patients to their bed as safe as possible, we designed and built the system to remind the patients ‘*pre*-*transfer tasks*’, to put on the brakes and raise the footrest of the wheelchair, as it is shown in Figure 3.

We used a touch sensor (HTSW, Sensatec), mounted on a bedrail, a tilt sensor (RBS32, ONCQUE), mounted on a brake and a force sensor (FSR402, INTERLINK) mounted on a footrest of the wheelchair. The actuators are voice alarming, neck muscle vibration and LEDs illumination on the top of the brake of the wheelchair. The case of stimulation for neck muscle vibration is shown in Figure 3.

Two transmitters (XBee RF modules, IEEE 802.15.4 RF modules by Digi International Inc.) are used to send the signals in a wireless manner from the three sensors to the receiver (XBee RF module). The output part produces the stimuli (voice, vibration, and LEDs illumination).

Tilt and force sensors are used to get the status of the brake and the footrest of the wheelchair. Based on the fact that the brake lever of a wheelchair is inclined when the patient puts on the brake, a tilt sensor finds the status of the brake. Similarly, a force sensor mounted on the footrest finds the status of the patient’s foot, i.e. whether the patient’s foot is on the footrest or not. The touch sensor is used as a target input to discover the intention of the patient (whether he tries to get off his foot to the floor before transferring to his bed or not).

**Figure 4:**
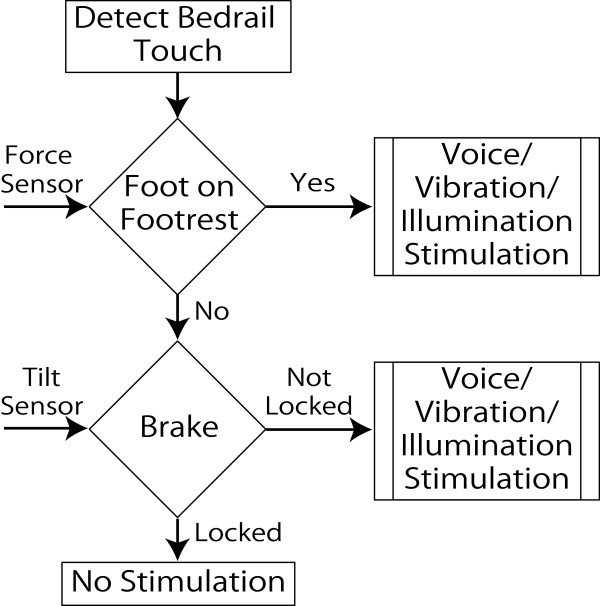
Stimulations flowchart.

The programmed logical arithmetic is shown in Figure 4. The order of the algorithm is in such a way that it first checks the status of the target input, i.e. the touch of the bedrail. Then, it checks for the statuses of the tilt and force sensors mounted on the brake and footrest, respectively. When the patient puts on the brake and raises the footrest of the wheelchair, the statuses of the sensors will be ‘Clear’ and the patient is provided with no stimulation. In the case that the statuses of the sensors are ‘not clear’, a voice or neck muscle vibration or LEDs illumination stimulation is produced. For the neck muscle vibration stimulation, the actuator vibrates the patient’s neck muscle. LEDs mounted on top of the brake illuminate for light stimulation. Multiple outputs of the system are also possible. For example, the combination of neck muscle vibration with LEDs illumination stimuli is possible.

Informed consents were obtained from two hemiplegic patients with hemispatial neglect whom participated in the verification of the system. These patients used to forget to put on breaks and raise the footrest before transferring from their wheelchairs to their beds. We included the pre-transfer tasks into our protocol for the assessment of the system. The system’s assessment criterion was: whether patients perform the pre-transfer tasks after stimulus (for both voice and neck muscle vibration stimulations), whether patients turn their heads towards the half paralyzed side of their body (for both voice and neck muscle vibration stimulations), and whether patients try to observe their paralyzed arm or leg after stimulus. We did not perform any neurophysiological assessment of patients’ brain before and after the rehabilitation because the main purpose of the system was its instant alerting effect on patients. In other words, we verified whether the patients’ actions with respect to the stimulus were similar to those that we expected to observe. The reason for this is because our focus was the practical usage of the system. This study and the procedure of the experiment were approved by the local Ethics Committee of the Nanakuri Sanatorium of Fujita Health University where the study took place.

Voice alarming stimulation was applied in the case of one patient and neck muscle vibration stimulation in the case of the other. The content of the voice alarming stimulation was ‘to put on the brakes and raise the footrests’. We could verify in both cases that the application of these stimuli helped patients to carry out the pre-transfer tasks before transferring to their beds.

## Results

During the transfer of the patient, we video-taped the transfer while we also checked to see if the system made the patient to remember to carry out the pre-transfer tasks. Other than these checking points of the system, we also asked the patients after rehabilitation sessions whether they could hear/feel the stimulus. In both cases, we could observe that the system effectively alarmed the patient to carry out the pre-transfer tasks before transferring to their beds. The patients expressed their satisfaction with the system saying that the alarming system made them not to forget the pre-transfer tasks and helped them to remember to take care of their paralyzed body space.

## Discussion

Vibrating the posterior neck musculature unilaterally makes an illusion in the healthy subjects that the head and stationary targets are displaced contralaterally [11,12]. Tachistoscopical stimulation in the contralesional left visual field together with patients’ left posterior neck muscle vibration application by Karnath et al. could result in inattention reduction in patients.

Robertson et al. [23] in another study could verify that an auditory tone sounded before a left visual stimulus can improve the awareness of the left visual field in the stroke patients with unilateral spatial neglect. Many studies [20,23,24] applied scanning training methods for the treatment of hemispatial neglect with significant improvements.

By learning from the previous studies and through our observation of inattention disorder in patients, we realized that a single approach/method can not assist all of the patients. Therefore, a multi-input/output system was designed and developed.

The applications of voice alarming stimulation and neck muscle vibration were promising in case of tests with two patients with hemispatial (sensory) neglects. However, this system and methodology can also be used with patients suffering from motor sensory neglect who underutilize one side of their body in spite of its normal strength and dexterity [29-32]. It is reported [30] that simple linguistic encouragement improves the performance of the ‘neglected’ hand. Therefore, the voice stimulation of our system can be used for these patients.

Another feature of our system is the application of these stimuli combined with the wheelchair that the patient uses. This has twofold effects in the rehabilitation of the patients. While the patients practice their activity of daily living task such as the transfer from their wheelchairs to their beds, skill enhancement can be achieved through true task repetition and the rehabilitation of the patients’ inattention can also be done.

Since the system is based on the wireless communication of transmitters and receiver covering a range of up to 30 meters, the functionality of the system is not restricted just to the patient’s room. The system functionalities can be expanded further. For example, patients suffering from inattention caused by higher cortical function disorder are in the risk of falling when they try to stand from the wheelchair, e.g., when the patients try to use a vending machine in a hospital; or when they try to transfer to a toilet. With some modifications of the algorithms in the receiver part and adding some more inputs (from sensors), the voice alarming stimulation can be used to remind all patients suffering from inattention disorder readily in the area of the hospital so that they avoid unexpected unsafe situations.

## Conclusion

A multi-input/output system for the rehabilitation and training of patients with inattention disorder was designed and developed. We could verify that the multi-input/output feature of the system was indispensable through the application of voice alarming and neck muscle vibration stimuli to two hemiplegic patients with hemispatial neglect. The system can be used with some minor modification broadly for all groups of patients suffering from inattention disorder as an alarming device for the daily living activities.

## Competing interests

The authors declare that they have no financial and non-financial competing interests that might be perceived to influence the results and/or discussion reported in this article.

## Authors’ contributions

AB was responsible for the design and development of the system. He carried out the experiments and drafted the manuscript. SO was involved with the design of the system. She financially supported the project and dealt with the medical issues related to the patients and the project. She supervised the trials with the patients. YT was involved with the design of the system and with the trials with the patients at the beginning. He approved the final draft. HM assisted to find suitable patients and helped with the set-up of the trials with patients. GT assisted with the set-up of the trials with patients. SS was involved with the design of the system. He supervised the whole project and approved the final draft. All authors read and approved the final manuscript.
